# 

**Published:** 1878-08

**Authors:** 


*Conventions—Associations—Recreation.*—By the time this number reaches the reader, the Pennsylvania State Dental Society will have held its Annual Meeting, at Bedford Springs, Pa., and the American Dental Association, the Southern Dental Association and the American Dental Convention will be in respective session at Niagara. Now that excursion tickets are sold so very cheaply, most, even of the poorer practitioners can afford to attend these meetings, and all will find them interesting and profitable. The vacation of even a week's absence from office labor will be beneficial, and the best thought of the profession is crystallized at these meetings. If occasionally there is wordiness and vagueness, remember that these speakers do not often get a chance to let out their accumulations, and there is much said that is good. To meet the representative men of the profession from all over the country, tends, as hardly anything else does, to broaden and elevate. Said a distinguished clergyman to the writer last year: "I am surprised at what I saw and heard at your convention. I had no idea that your profession as compared with others, was so scientific and of so cultivated an aspect. I think it is on a level with the highest; and I am proud to see so learned a body of men come together to discuss means for relieving human suffering." It is to be hoped that means may be set on foot to more thoroughly organize and consolidate the profession, and add to its *esprit de corps.* And it it is well for those of the State Associations who are interested in their home societies, to go to these central gatherings and study their workings. We are sure that few come from these meetings without gain. H.

---

### MONTHLY SUMMARY.

---

*Rules for Chloroform Administration.*—Prof. Syme had given chloroform, he said, for many years without an accident. He attributed his success to two causes. First, he always used good chloroform, and second, he always gave plenty of it.

### To Readers and Correspondents.

Communications intended for publication, and exchange journals and papers, should be addressed to the EDITOR OF THE AMERICAN JOURNAL OF DENTAL SCIENCE, 259 N. Eutaw St., Baltimore, Md. All letters relating to the business of the journal, or containing cash remittances, to be directed to Messrs. SNOWDEN & COWMAN. Articles for publication should be received by the editor at least three weeks previous to the first day of the month on which the number in which it is designed for them to appear, is issued.

### MONTHLY SUMMARY.

☞ The American Journal of Dental Science will contain fifty pages of reading matter in each number, making a volume of about

*Six Hundred* pages

EXCLUSIVE OF ADVERTISEMENTS

THE

### AMERICAN JOURNAL

OF

### DENTAL SCIENCE.

The *Journal* is issued on the first of each month and contains not less than *fifty* pages of reading matter in each number, exclusive of advertisements, making a volume of *six hundred* pages per annum.

In each number will be found Original Communications, Correspondence, Selected Articles, Monthly Summary, Bibliographical Notices and Editorial Matter, and every effort will be exerted to render the *Journal* worthy of the patronage of the Dental profession.

A generous co-operation is respectfully solicited from the members of the profession; and as the *Journal* has now a printing office of its own, which materially lessens the cost of its publication, it will be issued at the reduced subscription price of

**$2.50 Per Annum in Advance.**

Communications are respectfully solicited on all subjects pertaining to the practice of dentistry, as well as reports of cases occurring in dental practice.

### RATES OF ADVERTISING.

| | 1 MO. | 2 MO. | 3 MO. | 6 MO. | 12 MO. |
|---|---|---|---|---|---|
| 1 PAGE. | $15 00 | $22 50 | $30 00 | $50 00 | $100 00 |
| ½ " | 10 00 | 15 00 | 20 00 | 30 00 | 50 00 |
| ¼ " | 7 00 | 11 00 | 15 00 | 20 00 | 30 00 |
| ⅛ " | 5 00 | 8 00 | 11 00 | 15 00 | 20 00 |

All original communications designed for publication, works for review, new instruments for notice, exchanges, &c., should be directed to the editor, 259 N. Eutaw St., Baltimore, Md.

All advertisements and subscriptions should be addressed to

SNOWDEN & COWMAN,
*Publishers of the Am. Journal of Dental Science.*
86 W. FAYETTE ST. BET. CHARLES & LIBERTY STS.,
BALTIMORE, MD.

